# The association of ABO blood types with host susceptibility to haemorrhagic fever with renal syndrome

**DOI:** 10.1017/S0950268821002168

**Published:** 2021-09-29

**Authors:** Jing Li, Xuan Song, Xiangmao Bu, Yanzhen Wan

**Affiliations:** 1 Clinical Laboratory, Qingdao No. 6 People's Hospital, Qingdao 266033, China; 2 Clinical Laboratory, Qingdao Municipal Hospital, Qingdao 266011, China; 3 Clinical laboratory, Qingdao Women and Children's Hospital, Qingdao Women and Children's Hospital Affiliated to Qingdao University, Qingdao 266034, China

**Keywords:** ABO blood type, HFRS infection, susceptibility

## Abstract

Since the discovery of ABO blood types, there has been mounting evidence of the association between blood types and infectious diseases. However, so far, there is rarely available research about the potential role of ABO blood types in haemorrhagic fever with renal syndrome (HFRS) infection. Our aim was to investigate the relationship between ABO blood types and the development of HFRS infection in Qingdao, China. We carried out a retrospective study enrolling 116 HFRS patients as the case group and 373 healthy subjects as the control group. ABO blood type distribution was analysed using the Chi-square test and logistic regression analysis. Results showed that the distribution of ABO blood types between the two groups was significantly different (*X^2^* = 18.151, *P* < 0.05). Blood type B was less frequently observed [odds ratio (OR), 0.404; confidence interval (CI), 0.238–0.684; *P* < 0.01], while blood type AB was more frequently observed in the case group (OR, 2.548; CI, 1.427–4.549; *P* < 0.01). Since significantly more males were affected than females, we further analysed the data by gender as well as blood types and obtained consistent results for males. Our findings indicated that populations with blood type AB might be more prone to HFRS infection, whereas those with blood type B might be less susceptible to HFRS infection, which will help to make risk stratification in infection control.

## Introduction

Haemorrhagic fever with renal syndrome (HFRS) is one of the two emerging zoonotic diseases caused by hantavirus infection, which is characterised by fever, haemorrhagic manifestations and renal dysfunction [1]. It is mainly prevalent across Europe and Asia, and China accounts for 90% of all HFRS cases worldwide [2]. HFRS cases have been reported in all 31 Chinese provinces in Mainland China. A total of 166 975 HFRS cases and 1689 HFRS-related deaths were reported in 31 provinces between 2004 and 2016 [3]. Although environment management, host surveillance and HFRS vaccine implementation have played an important role in controlling HFRS, it is still a serious disease in Mainland China.

Human ABO blood types are defined by genetically derived glycoconjugate structures specific to the antigens that are located on the red cell surface [4]. The relative distribution of ABO types can vary among different ethnic populations, although group O tends to be the most common. Since the discovery of ABO blood types, there has been an ongoing interest in the association between blood types and different infectious diseases [5, 6]. Early aetiological studies indicated that blood type O has a connection with increased incidence of cholera, plague, tuberculosis infections and mumps, whereas blood type A is linked with increased incidence of smallpox and *Pseudomonas aeruginosa* infection; blood type B is associated with increased incidence of gonorrhoea, tuberculosis and *Streptococcus pneumoniae*, *Escherichia coli* and salmonella infections; and blood type AB is associated with increased incidence of smallpox and *E. coli* and salmonella infections [7].

However, so far, there is rare available research about the potential role of ABO blood types in HFRS infection. Here, we studied the relationship between ABO blood types and the development of HFRS infection in Qingdao, China.

## Methods

### Study population and data collection

This retrospective study enrolled 116 HFRS patients admitted in Qingdao No. 6 People's Hospital from Feb 2015 to Oct 2020 as the case group. They were aged from 6 to 80 years old with an average age of 49, and 91 cases (78.45%) were male and 25 cases (21.55%) were female. Eighty-one cases (69.83%) were farmers. Meanwhile, 373 healthy subjects for physical examination in the hospital during the according time were selected as the control group. They were aged from 3 to 81 years old with an average age of 34, and 217 (58.18%) were male and 156 (41.82) were female. All the included HFRS patients were confirmed according to the HFRS diagnostic standards (WS278-2008) including epidemiological history, clinical symptoms and positive anti-hantavirus IgG/IgM.

### ABO and RhD blood type identification

Peripheral venous blood samples were collected in ethylene diamine tetraacetic acid (EDTA)-containing vacuum blood collection tubes. ABO and RhD blood types were determined using a blood type gel card (Ortho-Clinical Diagnostics, the United Kingdom). Forward and reverse typing were both performed.

### Statistical analysis

Data analysis was performed using SPSS 17.0. ABO blood type distribution between two groups was analysed using Chi-square test and logistic regression. *P* < 0.05 (two-tailed) was considered to be statistically significant.

## Results

### Gender and age distribution of 116 HFRS patients

As is shown in Table 1, of all the HFRS patients, 91 cases (78.45%) were male and 25 cases (21.55%) were female. More than three times as many men were affected as women. They were aged from 6 to 80 years old, with the average age of 49 [two cases (1.72%), aged >5–⩽17; 16 cases (13.79%), aged >17–⩽34; 61 cases (52.59%), aged >34–⩽55; 37 cases (31.90%), aged >55]. Obviously, middle-aged and aged people accounted for most of the patients.

**Table 1.** Gender and age distribution of HFRS patients

Characteristics	Number (*n*)	Percentage (%)
Gender		
Male	91	78.45
Female	25	21.55
Age		
>5–⩽17	2	1.72
>17–⩽34	16	13.79
>34–⩽55	61	52.59
>55	37	31.90

### Distribution of ABO and RhD blood types between two groups

ABO distribution in the case group was as follows: A (33.62%), B (17.24%), O (29.31%) and AB (19.83%). ABO distribution in the control group was A (30.83%), B (34.05%), O (26.27%) and AB (8.85%), which was consistent with that reported for the local population. The distribution of ABO blood types between the two groups was significantly different (*X^2^* = 18.151，*P* < 0.05) (Table 2). As regards RhD status, all individuals in the case group and in the control group were RhD positive.

**Table 2.** Distribution of ABO blood types between two groups

	A	B	O	AB
Control group (*n* = 373)	115（30.83%）	127（34.05%）	98（26.27%）	33（8.85%）
Case group (*n* = 116)	39（33.62%）	20（17.24%）	34（29.31%）	23（19.83%）

*X^2^* = 18.151, *P* < 0.05.

### The association between ABO blood types and the likelihood of HFRS infection

To evaluate the role of ABO blood types in the likelihood of HFRS infection, logistic regression analysis was performed. Results showed that blood type B was less frequently observed in the case group, compared with the control group [odds ratio (OR), 0.404; confidence interval (CI), 0.238–0.684; *P* < 0.01]. Conversely, blood type AB was more frequently observed in the case group (OR, 2.548; CI, 1.427–4.549; *P* < 0.01). No significant difference in blood type A and O was found between the two groups (Table 3). More males were affected than females, so we further analysed the data by gender as well as blood types. As is demonstrated in Table 4, for males, likewise, blood type B was less frequently observed (OR, 0.448; CI, 0.243–0.825; *P* < 0.01), while blood type AB was more frequently observed in the case group (OR, 2.339; CI, 1.196–4.574; *P* < 0.05). For females, though blood type B distribution was lower and blood type AB distribution was higher in the case group, no significant difference was found (Table 4).

**Table 3.** Odds of HFRS infection according to ABO blood types

	A	B*	O	AB*
Control group	115（30.83%）	127（34.05%）	98（26.27%）	33（8.85%）
Case group	39（33.62%）	20（17.24%）	34（29.31%）	23（19.83%）
OR	1.136	0.404	1.164	2.548
95% CI	0.729–1.771	0.238–0.684	0.733–1.846	1.427–4.549
*P* value	0.572	0.001	0.520	0.001

OR, odds ratio; CI, confidence interval; * *P* < 0.05 was considered to be statistically different.

**Table 4.** Odds of HFRS infection according to gender as well as ABO blood types

	Male				Female			
	A	B*	O	AB*	A	B	O	AB
Control group	60（27.65%）	70（32.26%）	65（29.95%）	22(10.14%)	55（35.26%）	57（36.54%）	33（21.15%）	11(7.05%)
Case group	28（30.77%）	16（17.58%）	28（30.77%）	19(20.88%)	11（44.00%）	4（16.00%）	6（24.00%）	4(16.00%)
OR	1.163	0.448	1.039	2.339	1.443	0.331	1.177	2.511
95% CI	0.681–1.987	0.243–0.825	0.611–1.768	1.196–4.574	0.613–3.394	0.108–1.012	0.435–3.184	0.732–8.611
*P* value	0.580	0.009	0.887	0.011	0.399	0.044	0.748	0.132

OR, odds ratio; CI, confidence interval; * *P* < 0.05 was considered to be statistically different.

## Discussion

Blood group antigens represent polymorphic traits inherited among individuals and populations. Differences in blood group antigen expression can increase or decrease host susceptibility to many infectious diseases [4, 8]. Here, we presented the relationship between ABO blood types and host susceptibility to HFRS infection.

In this study, we found that the majority of HFRS cases were middle-aged and aged male farmers. More than three times as many males were affected as females, and middle-aged and aged people accounted for most of the patients, which is consistent with the surveillance report from 2004 to 2016 in China [3]. Farmers are more likely to work in rodent-infested areas such as fields and barns and are exposed to rodent urine, faeces and saliva. In addition, middle-aged and aged males compose the main part of farmers in China, which might explain the reason why they are the most susceptible to HFRS infection.

In recent years, accumulating evidence has been found to support the important role of blood type in the development of different infectious diseases including bacterial infections and viral infections. For example, since the emerging of coronavirus disease-2019 (COVID-19) infection worldwide, the relationship between ABO blood types and COVID-19 infection rate, symptom presentation and outcome has been researched in a number of studies [9, 10]. Key findings suggested that blood type O might serve as a protective factor, as individuals with blood type O were found to be COVID-19 positive at far lower rates, although there were also inconsistent results [11, 12]. In this study, our findings showed that blood type B was less frequently observed, while blood type AB was more frequently observed in HFRS infection, indicating that populations with blood type AB might be more prone to HFRS infection, whereas those with blood type B might be less susceptible to HFRS infection. Since significantly more males were affected than females, we further analysed the data by gender as well as blood types and obtained consistent results for males. For females, though blood type B distribution was lower and blood type AB distribution was higher in the case group, no significant difference was found between two groups, which might be caused by insufficient sample size. To our knowledge, this is the first report about the relationship between ABO blood types and host susceptibility to HFRS infection, even though these results should be considered preliminary. As for RhD blood group, it was reported that only 1.02% of participants were RhD negative in China [13]. And in this study, all individuals in the case group and in the control group were RhD positive, so we cannot make any conclusion about whether RhD status could affect HFRS infection, which is inherent to the studied populations. Further studies in populations with a higher proportion of RhD negative individuals might address this problem more definitively.

Blood type antigens may be involved in infection directly or indirectly [5]. They can serve as receptors and /or co-receptors for toxins, bacteria and viruses, where they facilitate intracellular uptake, signal transduction and adhesion through the organisation of membrane microdomains. Blood type antigens can also serve as false receptors to inhibit binding to target cells [14]. In addition, several blood types can modify the innate immune response to infection. Anti-A and anti-B antibodies can be considered part of the innate immune system against several infectious agents carrying A and B motifs [15]. Of more significance is the finding that blood group antigens and secretor status are genetically determined host factors that influence the composition of the human intestinal microbiome, which plays a vital role in health and disease, beginning in the prenatal period and extending throughout childhood [16]. Hantaviruses are a family of enveloped single-stranded RNA viruses, hosted mainly by small rodents and spread to people via an aerosolised virus that is shed in urine, faeces and saliva and less frequently by a bite from an infected host, causing HFRS and hantavirus pulmonary syndrome (HPS) [17]. Hantaviruses infect endothelial, macrophage, epithelial, follicular dendritic and lymphocytic cells by means of the attachment of viral glycoprotein with host cell surface receptors [18]. The exact mechanisms by which ABO blood types affect host susceptibility to HFRS infection remain to be further investigated, although the above mentioned might be involved.

Our study has several limitations. It is a single-centre, retrospective, observational study with a relatively small sample size. We could not subcategorise blood types by Rhesus factor positivity and did not analyse clinical outcomes among different ABO blood types because of small sample size. Further research of the mechanistic link between ABO antigens, antibodies and HFRS infection and severity, and its implications on controlling the current epidemic is warranted. So far, no specific treatment or vaccines have been approved by the U.S. Food and Drug Administration to treat or prevent hantavirus-caused syndromes. Better understanding of the association between ABO blood types and HFRS infection will help to develop prophylactic and therapeutic strategies for HFRS, as well as to make risk stratification in infection control.

## Data Availability

The datasets used and/or analysed in the current study are available from the corresponding author on reasonable request.
